# Identification of two novel mammographic density loci at 6Q25.1

**DOI:** 10.1186/s13058-015-0591-2

**Published:** 2015-06-03

**Authors:** Judith S Brand, Jingmei Li, Keith Humphreys, Robert Karlsson, Mikael Eriksson, Emma Ivansson, Per Hall, Kamila Czene

**Affiliations:** Department of Medical Epidemiology and Biostatistics, Karolinska Institutet, Nobels Väg 12A, 171 77 Stockholm, Sweden; Swedish eScience Research Centre (SeRC), Karolinska Institutet, Nobels Väg 12A, 171 77 Stockholm, Sweden

## Abstract

**Introduction:**

Mammographic density (MD) is a strong heritable and intermediate phenotype for breast cancer, but much of its genetic variation remains unexplained. We performed a large-scale genetic association study including 8,419 women of European ancestry to identify MD loci.

**Methods:**

Participants of three Swedish studies were genotyped on a custom Illumina iSelect genotyping array and percent and absolute mammographic density were ascertained using semiautomated and fully automated methods from film and digital mammograms. Linear regression analysis was used to test for SNP-MD associations, adjusting for age, body mass index, menopausal status and six principal components. Meta-analyses were performed by combining *P* values taking sample size, study-specific inflation factor and direction of effect into account.

**Results:**

Genome-wide significant associations were observed for two previously identified loci: *ZNF365* (rs10995194, *P* = 2.3 × 10^−8^ for percent MD and *P* = 8.7 × 10^−9^ for absolute MD) and *AREG* (rs10034692, *P* = 6.7 × 10^−9^ for absolute MD). In addition, we found evidence of association for two variants at 6q25.1, both of which are known breast cancer susceptibility loci: rs9485370 in the *TAB2* gene (*P* = 4.8 × 10^−9^ for percent MD and *P* = 2.5 × 10^−8^ for absolute MD) and rs60705924 in the *CCDC170*/*ESR1* region (*P* = 2.2 × 10^−8^ for absolute MD). Both regions have been implicated in estrogen receptor signaling with TAB2 being a potential regulator of tamoxifen response.

**Conclusions:**

We identified two novel MD loci at 6q25.1. These findings underscore the importance of 6q25.1 as a susceptibility region and provide more insight into the mechanisms through which MD influences breast cancer risk.

**Electronic supplementary material:**

The online version of this article (doi:10.1186/s13058-015-0591-2) contains supplementary material, which is available to authorized users.

## Introduction

Mammographic density (MD) reflects the amount of radiographically dense tissue on an X-ray of the breast (mammogram) and is an intermediate phenotype for breast cancer [1]. MD is highly heritable (*h*^*2*^ = 0.60 – 0.65) [2–5] and genetic loci associated with MD can provide insight into the biological mechanisms leading to breast cancer, which may serve as targets for treatment and preventive strategies [6]. Despite the high heritability, a large proportion of the genetic variation of MD remains unexplained [7–9]. The Marker of Density (MODE) consortium recently identified nine loci (*AREG*, *ESR1*, *ZNF365*, *LSP1/TNNT3*, *IGF1*, *TMEM184B*, *SGSM3/MKL1*, *PRDM6*, 8p11.23) associated with area-based MD [7, 8] as obtained with the semiautomated thresholding method Cumulus [10]. Although Cumulus is still regarded as the ‘gold standard’ for screen-film mammography, fully automated methods may help in the identification of additional variants as these methods are less prone to random measurement error. We performed a large-scale genetic association study combining semiautomated and fully automated density measures to identify novel MD loci.

## Methods

### Study participants

For the present study, we included participants of European ancestry from three Swedish studies: KARolinska MAmmography project for risk prediction of breast cancer (KARMA), Linné-bröst 1 (LIBRO-1) and the Singapore and Sweden Breast Cancer Study (SASBAC). KARMA is a prospective screening-based study initiated in January 2011 and includes 70,877 women who attended mammography screening or clinical mammography at four hospitals in Sweden. In 2010, a random sample of 5,531 cancer-free women was genotyped of whom 4,025 had raw digital mammograms stored. LIBRO-1 is a breast cancer cohort including 5,715 malignant cases diagnosed between 2001 and 2008 in the Stockholm/Gotland area. The majority of the cohort (N = 5,125) was genotyped and prediagnostic film mammograms were successfully retrieved for 2,805 women. A further 1,589 women were drawn from the SASBAC study, which is a population-based case–control study including postmenopausal breast cancer cases in Sweden aged 50 to 74 years at time of enrollment (1 October 1993 to 31 March 1995) and age-matched controls. Ethical approval of KARMA, LIBRO-1 and SASBAC was given by the ethical review board at Karolinska Institutet (Stockholm, Sweden) and written informed consent was obtained from all participants.

### Assessment of mammographic density

Mammographic density was obtained from the mediolateral oblique (MLO) view in all three studies using different measurement tools. In KARMA, MD was estimated from raw digital mammograms using a volumetric method (Volpara) [11]. Volpara shows good agreement with breast magnetic resonance imaging (MRI) data [11] and its measures (percent and absolute dense volume) have been validated as being predictive of breast cancer risk [12, 13]. In SASBAC and LIBRO-1, MD was estimated using an area-based method from film mammograms with respectively Cumulus [10] and an automated algorithm based on the image processing software ImageJ that mimics Cumulus [14, 15]. ImageJ shows good agreement with Cumulus (the ‘gold standard’ for film mammography) with high levels of correlation for both percent and absolute dense area [13, 16, 17].

Since volumetric measures incorporate information on breast thickness, the underlying distribution of area-based and volumetric measures are slightly different, with the latter being more right-skewed with a smaller range of possible values (Figure S1 in Additional file 1).

### Genotyping and imputation

All women were genotyped using the custom Ilumina iSelect genotyping array of the Collaborative Oncological Gene-environment Study (iCOGS) which comprises 211,155 single nucleotide polymorphisms (SNPs) primarily selected for replication of loci putatively associated with breast cancer and other cancers [18]. Details of the iCOGS array design, sample handling and post-genotyping quality control (QC) processes are described in depth elsewhere [18]. In brief, samples were excluded from analysis for any of the following reasons: low or high heterozygosity, individuals not concordant with previous genotyping, discordant duplicate pairs and first-degree relatives. Standard SNP QC was performed in Plink (version 1.07) [19] and SNPs with minor allele frequency (MAF) <0.01 or deviation from Hardy–Weinberg equilibrium (HWE) at *P* <1 x 10^−6^ in controls or *P* <1 x 10^−12^ in cases were excluded, leaving 170,798 SNPs for the combined analyses. To increase resolution and coverage for regional association testing, nongenotyped SNPs were imputed using the 1000 Genomes Project March 12 release as a reference [20]. Data were imputed in a two-stage procedure, using SHAPEIT to derive phased genotypes and IMPUTE version 2 (IMPUTEv2) to perform the imputation on the phased data [21]. The imputation was performed using 5 Mb nonoverlapping windows across the whole genome. Postimputation quality control was based on the IMPUTE info score and SNPs with a score ≤0.80 or MAF <0.01 were excluded.

### Statistical analyses

SNP association analysis was performed separately within each study. Genotyped SNPs were analysed in Plink (version 1.07) [19] using linear regression and assuming an additive genetic model. We analyzed three MD phenotypes: percent density (percent MD), absolute dense tissue (absolute MD) and the absolute nondense tissue. Since volumetric mammographic measures follow a different distribution than area-based measures, different types of transformation were used to approximate the normal distribution (log-transformation for volumetric measures and square-root transformation for area-based measures) (Figure S1 in Additional file 1).

Differences in study design and measurement technique did not allow us to perform meta-analyses based on study-specific effect estimates (beta coefficients). Instead, we performed meta-analyses combining study-specific *P* values in METAL (25 March 2011 release) [22] taking sample size, study-specific inflation factor and direction of effect into account.

Regional association plots were generated using LocusZoom with the 400 kb region centered on the index SNP [23]. Imputed SNPs within the region were analyzed with SNPTEST (version 2.5.2) [24, 25] based on the score test, which uses allele dosages instead of genotype calls.

Population stratification was assessed using principal component (PC) analysis in EIGENSTRAT (version 3.0) [26, 27]. All analyses were adjusted for age (years), body mass index (BMI) (kg/m^2^), menopausal status (postmenopausal vs premenopausal) and six study-specific PC scores to account for population substructure.

### Functional annotation and breast cancer association analysis of identified variants

Functional annotation of associated variants and their proxies (r^2^ ≥0.8 in 1000 Genomes CEU population) was performed using the HaploReg v2 software [28]. We studied putative functional variants using data from the Encyclopedia of DNA Elements (ENCODE) project [29], in particular the chromatin state segmentation [30] for the human mammary epithelial cells (HMEC) cell line.

We checked for associations between associated variants and breast cancer risk by doing a lookup in the Breast Cancer Association Consortium (BCAC) including a total of 55,540 breast cancer cases and 51,168 controls with iCOGS genotyping data. We also verified associations with MD loci that were previously identified by the genome-wide association study (GWAS) coordinated by MODE [8]. These analyses were performed in KARMA and LIBRO-1 participants only, as SASBAC is part of the MODE consortium.

## Results

Table 1 summarizes the characteristics of the study participants in each individual study. Most participants were postmenopausal: 76.1 % in LIBRO-1 and 100 % in SASBAC, with a mean age of 58.4 years and 62.4 years respectively. Participants of KARMA were slightly younger (mean age = 53.6 years), with a larger contribution of premenopausal women (51.0 %). No substantial difference in BMI was observed across the individual studies.

**Table 1:** Descriptive characteristics of the studies included

Study	Number	Mammogram	Measure	Age (years)	BMI (kg/m^2^)	Post-menopause	Percent density (%)^a^	Absolute dense^a^	Absolute nondense^a^
				Mean (SD)	Mean (SD)	Percent (N)	Median (IQR)	Median (IQR)	Median (IQR)
KARMA	4025	Raw digital	Volpara	53.6 (9.4)	25.3 (4.2)	51.0 (2,054)	8.4 (6.5)	60.4 (36.8)	677 (581)
SASBAC	1589	Digitized screen-film	Cumulus	62.4 (6.4)	25.6 (3.8)	100 (1,589)	11.8 (17.7)	18.1 (26.2)	140 (76)
LIBRO-1	2805	Digitized screen-film	ImageJ	58.4 (8.8)	25.3 (4.0)	76.1 (2,134)	30.0 (21.6)	30.6 (21.5)	71 (30)

All mammograms were from the mediolateral oblique (MLO) view

*BMI* body mass index, *SD* standard deviation, *IQR* interquartile range

^a^Percent density (percent MD) in %; absolute dense tissue (absolute MD) in cm^3^ (KARMA) and cm^2^ (SASBAC and LIBRO-1); absolute nondense tissue in cm^3^ (KARMA) and cm^2^ (SASBAC and LIBRO-1)

Quantile-quantile (QQ) plots for each MD phenotype are shown in Figure S2 in Additional file 2.

All plots displayed no global departure from the expected null distribution of *P* values and the genetic inflation factor (λ) was 1.005, 1.015 and 1.024 for KARMA, LIBRO-1 and SASBAC respectively, indicating that residual confounding by population stratification is negligible.

**Fig. 1:**
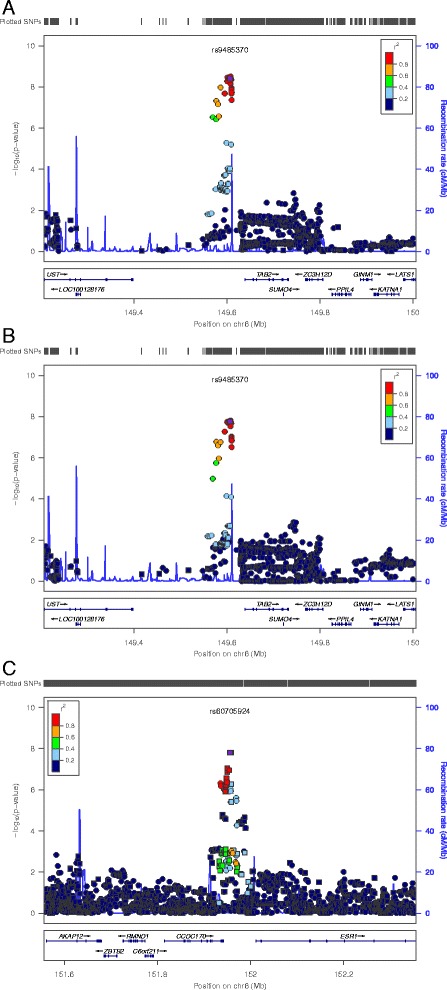
Regional plots of SNPs associated with percent and absolute mammographic density. **a** = regional association plot rs9485370 for percent density; (**b**) = regional association plot rs9485370 for absolute dense tissue; (**c**) = regional association plot rs60705924 for absolute dense tissue. Plot shows –log10 *P* values (y-axis) by chromosomal position (x-axis). Top genotyped SNPs (rs9485370 and rs60705924) are shown in purple. Squares denote genotyped SNPs; circles denote imputed SNPs. Colors indicate the extent of linkage disequilibrium with rs9485370 and rs60705924. Genetic recombination rates are estimated using 1000 Genomes EUR sample and are shown with the light blue line. Physical positions are based on NCBI build37 of the human genome. Note: rs9485370 falls within transcript ENST00000536230 of TAB2, which spans chr6:149539777–149731075. The plot was generated using LocusZoom software. *SNP* single nucleotide polymorphism

Figure S3 (Additional file 3) shows the Manhattan plots displaying the log10-transformed *P* values for each genotyped SNP per MD phenotype. In total, we identified two loci for percent MD (*TAB2, ZNF365*) and four loci for absolute MD *(AREG, TAB2, CCDC170/ESR1, ZNF365)* (Table 2; Fig. 1). Two of the loci *(ZNF565, AREG)* were recently identified by MODE [7, 8], but the loci mapping to 6q25.1 (*TAB2* and *CCDC170/ESR1)* have not been reported previously as being associated with MD at a genome-wide significance level. No significant associations were observed for the absolute nondense tissue (Figure S3 in Additional file 3).

**Table 2:** Single nucleotide polymorphisms associated with percent and absolute mammographic density

					KARMA			SASBAC			LIBRO-1				
CHR	SNP	BP	Genes	Alleles^a^	MAF	beta (se)	*P*	MAF	beta (se)	*P*	MAF	beta (se)	*P*	*P* overall	*P* het
Percent density															
6	rs9485370	149606801	*TAB2*	G/T	0.18	−0.05 (0.01)	2.3 × 10^−5^	0.17	−0.16 (0.08)	0.04	0.17	−0.16 (0.05)	3,9 × 10^−4^	4.8 × 10^−9^	0.88
10^b^	rs10995194	64288130	*ZNF365*	G/C	0.16	−0.05 (0.01)	7.1 × 10^−6^	0.15	−0.13 (0.08)	0.10	0.15	−0.15 (0.05)	1.9 × 10^−3^	2.3 × 10^−8^	0.62
Absolute dense															
4^b^	rs10034692	75419787	*AREG*	A/G	0.32	−0.04 (0.01)	9.6 × 10^−5^	0.31	−0.23 (0.08)	6.1 × 10^−3^	0.31	−0.14 (0.04)	5.5 × 10^−4^	6.7 × 10^−9^	0.96
6	rs9485370	149606801	*TAB2*	G/T	0.18	−0.04 (0.01)	2.0 × 10^−3^	0.17	−0.22 (0.10)	0.03	0.17	−0.22 (0.05)	1.0 × 10^−5^	2.5 × 10^−8^	0.37
6	rs60705924	151955985	*CCDC170-ESR1*	A/G	0.31	0.04 (0.01)	1.9 × 10^−4^	0.31	0.33 (0.08)	3.0 × 10^−5^	0.31	0.09 (0.04)	0.03	2.2 × 10^−8^	0.13
10^b^	rs10995194	664288130	*ZNF365*	G/C	0.16	−0.07 (0.01)	1.1 × 10^−6^	0.15	−0.21 (0.10)	0.05	0.15	−0.15 (0.05)	5.1 × 10^−3^	8.7 × 10^−9^	0.55

Genes refer to genes and nearby genes

*MD* mammographic density, *CHR* chromosome, *SNP* single nucleotide polymorphism, *BP* base pair position (NCBI Build 37), *MAF* minor allele frequencies; *P* overall = *P* value meta-analysis; P het = *P* value chi-square test for heterogeneity

^a^Major allele (reference allele)/minor allele (effect allele)

^b^Previously identified in the Marker of Density (MODE) consortium: R^2^ rs10995194 and MODE SNP rs10995190 = 1 (1000 Genomes Project, Pilot 1 (CEU))

The strongest association at 6q25.1 was found for rs9485370 in the TGF-beta-activated kinase 1/MAP3K7-binding (*TAB2*) gene, where each additional copy of the minor allele was associated with a decrease in percent MD (*P* = 4.8 × 10^−9^) and absolute MD (*P* = 6.7 × 10^−9^) (Table 2, Fig. 1). Rs9485370 is located in a putative enhancer element in HMEC cell lines and is linked to a number of SNPs which have been predicted to influence transcription factor binding (Figure S4 in Additional file 4)*.*

The minor allele frequency (MAF) of rs9485370 varies widely across populations with the effect allele (T) being more common in Asians than Europeans. Rs9485370 is not an established breast cancer SNP in women of European ancestry, but an SNP in complete linkage disequilibrium (LD) (rs9485372; r^2^ = 1, D’ = 1) has previously been associated with breast cancer risk in East Asian women [31, 32]. Rs9485370 did not reach genome-wide significance in BCAC, but there was evidence of a stronger association in Asian (odds ratio (OR) = 0.89, *P* = 7.4 × 10^−6^) than in European women (OR = 0.96, *P* = 1.4 × 10^−3^) and the direction of association was consistent with the effect of MD on breast cancer risk (Table S1 in Additional file 5). All associated SNPs in the 400 kb window were in LD with s9485370 and there was no evidence of additional independent signals in this locus (Fig. 1).

The second hit at 6q25.1 was rs60705924, located 14 kb downstream of *CCDC170* and 22 kb upstream of *ESR1*. Each minor allele at rs60705924 was associated with an increase in absolute MD (*P* = 2.2 × 10^−8^) (Table 2, Fig. 1) but the association was weaker and nonsignificant for percent MD *(P =* 1.2 × 10^−4^). Rs60705924 is strongly correlated with breast cancer SNP rs2046210 (r^2^ = 0.89, D’ = 1.00) [18] and its association with breast cancer (OR in BCAC European sample = 1.08, *P* = 1.9 × 10^−13^) follows the same direction as its association with absolute MD (Table S1 in Additional file 5). Three SNPs in strong LD with rs60705924 (rs7763637, rs6557160, rs6913578) map to promoter/enhancer histone marks in HMEC cell lines (Table S4 in Additional file 4). Rs60705924 is also in proximity to rs12665607, a SNP that was recently found to be associated with absolute dense area in MODE [8] and highly correlated with breast cancer SNP rs12662670 (r^2^ = 0.89; D’ = 1.00). However, rs60705924 and rs12665607 are only weakly correlated (r^2^ = 0.19; D’ = 1.00) and the association with rs60705924 was only slightly attenuated in conditional analysis (*P* = 4.8 × 10^−6^). Regional association analysis revealed no additional independent SNPs in the 400 kb window of rs60705924 (Fig. 1).

We also tested for associations with MD loci that were previously identified in GWAS coordinated by MODE. We could confirm associations with the majority of loci found by MODE (Table S2 in Additional file 6), except for *LSP1* (*P* = 0.25 for percent MD), *TMEM184B* (*P* = 0.14 for percent MD and *P* = 0.30 for absolute dense tissue) and rs7816345 at chromosome 8 (*P* = 0.31 for percent MD)*,* although there was some evidence of an association between *TMEM184B* and volumetric MD in KARMA. Although no SNPs reached genome-wide significance in our meta-analysis of absolute nondense tissue, we could replicate the nondense locus (rs7816345) that was recently identified by MODE (*P* = 2.4 × 10^−4^) (Table S2 in Additional file 6).

## Discussion

We performed a meta-analysis of three large-scale genetic association studies to identify novel MD loci. Using semiautomated and fully automated measures, we were able to identify two additional variants at 6q25.1 (*TAB2* and *CCDC170*/*ESR1*) that were associated with both volumetric and area-based MD. We also confirmed associations with several loci (*ZNF365* and *AREG*) that were previously identified by MODE [8].

Like MODE, we identified more genetic loci for absolute than for percent MD. Our most significant hit was rs9485370 mapping to the *TAB2* gene. This SNP has previously been associated with breast cancer risk in East Asian women [31, 32], but not with mammographic density at a genome-wide significance level. The protein encoded by the *TAB2* gene is an important mediator of interleukin-1 (IL-1)-induced activation of the NFkB and MAPK8/JNK pathway [33] which has been associated with early tumorigenesis and metastasis [34, 35] as well as mammary development [36]. The TAB2 protein also interacts directly with the N-terminal domain of the estrogen receptor alpha (ESR1) and has been implicated in proinflammatory induced reactivation of repressed estrogen receptor (ER) signaling pathways [37, 38]. Because of its role in ER signaling, TAB2 is seen as a potential target for reversing tamoxifen resistance in breast cancer cells [38].

The second variant at 6q25.1 (rs60705924) is located in *CCDC170*/*ESR1* region*,* a well-established breast cancer locus, but its putative functions are not well defined. Previous GWAS and candidate approaches have identified multiple genetic variants at *CCDC170*/*ESR1* to be associated with breast cancer as well as mammographic density [39]. A breast cancer SNP in strong LD with rs60705924 and rs2046210 has previously been identified in candidate approaches of area-based and volumetric MD [5, 7], but not at genome-wide significance level. SNP rs2046210 is more strongly associated with ER-positive than -negative tumors [40, 41] and our data suggest that at least part of the association with breast cancer is mediated through mammographic density. Recent data further indicate that recurrent rearrangements between the *ESR1* and *CCDC170* gene are linked to more aggressive and endocrine-resistant cancers [42]. Fine-mapping studies of 6q25.1 are needed to provide more insight into the independent and causal variants in this specific region.

To our knowledge, this is one of the largest studies analyzing genetic determinants of fully and semiautomated MD measures. All mammograms in the study were obtained from the MLO view and all participants were genotyped on the same genotyping platform, reducing the likelihood of measurement errors due to between-view and interassay differences. However, our findings need to be interpreted in light of the different MD methods used. First of all, we combined screen-film and digital mammograms in our meta-analysis. Previous studies have shown that MD measurements from digital mammograms tend to be lower than from film mammograms [43]. Furthermore, different measurement tools were used in each individual study. Both area-based and volumetric methods aim to quantify the amount of fibroglandular tissue in the breast from two-dimensional mammograms, but the measurement techniques used are slightly different. Area-based methods use an intrinsic threshold technique [either semiautomated (Cumulus) or fully automated (ImageJ)] to categorize pixels as dense or nondense, whereas Volpara is specifically designed to quantify the density in each individual pixel on a continuous scale while accounting for interindividual differences in breast thickness. Several studies have evaluated the agreement between Cumulus and ImageJ, all showing high correlation coefficients for percent dense area (*r* ranging from 0.88 to 0.92) [13, 16, 17] and absolute dense area (*r* ranging from 0.89 to 0.90) [13, 17]. High levels of agreement have also been reported for percent dense area and volume (*r* ranging from 0.86 to 0.93) [12, 13], and recent data confirm the good overall correlation between all three measures with similar breast cancer risk estimates for Cumulus, ImageJ and Volpara percent MD [13, 44]. The correlation between absolute dense area and volume is somewhat weaker (*r* ranging from 0.41 to 0.55) [12, 13]. Heritability estimates also tend to be lower for the absolute dense volume than for the absolute dense area [5], indicating that these absolute measures represent different aspects of MD. Since we cannot rule out the presence of area- and volumetric-specific MD loci, we could have missed SNPs that are associated with the absolute dense volume, but not with its area-based counterpart. This might also explain the lack of replication for some of the SNPs previously identified by MODE for area-based MD [8]. Future GWAS aimed at identifying genetic variants of both area-based and volumetric MD will provide more insight into this matter. Although our study might be limited in terms of power due to the combination of different MD methods (e.g., mammogram type and measurement technique), this could not have affected the validity of our findings. Of note, all SNP-MD associations were in the same direction with no evidence of between-study heterogeneity. As such, our study has identified loci that are associated with MD, regardless of measurement technique and mammogram type used.

Studies identifying MD loci are important to increase our understanding of the biological mechanisms leading to breast cancer in women with high mammographic density. Such insights come primarily from SNPs that are associated with both mammographic density and breast cancer risk [12, 45], including the variants identified in the present study. This information might also be relevant for identifying new targets for treatment and preventive strategies. Our results, for instance, highlight the importance of the 6q25.1 region in the etiology of breast cancer among women with dense breasts. From a clinical perspective, SNPs and downstream pathways that are associated with mammographic density, but not with breast cancer risk, are of limited value, as these SNP-MD associations are not likely to influence breast cancer as a disease endpoint.

## Conclusions

In conclusion, we identified two novel MD loci at 6q25.1 in a large-scale genotyping effort of semiautomated and fully automated MD measures, which have previously been associated with breast cancer risk. These findings underscore the importance of 6q25.1 as a susceptibility region and provide more insight into the mechanisms through which MD influences breast cancer risk. Future large-scale genetic association studies of area-based and volumetric MD are needed to increase our understanding of the genetic basis of mammographic density and its link with breast cancer.

## Additional files

Additional file 1: Figure S1. Distributions of mammographic density phenotypes, stratified by study. Distributions of mammographic measures before (A) and after transformation (B). Percent density in %; absolute dense tissue in cm^3^ (KARMA) and cm^2^ (SASBAC and LIBRO-1); absolute nondense tissue in cm^3^ (KARMA) and cm^2^ (SASBAC and LIBRO-1). Volumetric mammographic measures were log-transformed (KARMA) and area-based mammographic measures were square-root-transformed (SASBAC and LIBRO-1) prior to analyses. Additional file 2: Figure S2. Quantile-quantile (QQ) plots, per mammographic density phenotype. A = percent density; B = absolute dense tissue; C = absolute nondense tissue. The observed *P* values based the on meta-analysis of KARMA, SASBAC and LIBRO-1 are plotted against the expected distribution of *P* values under the null distribution. Additional file 3: Figure S3. Manhattan plots of the combined association results, per mammographic density phenotype. A = percent density; B = absolute dense tissue; C = absolute nondense tissue. The –log_10_ (*P*) values are plotted against chromosomal base-pair position. Genome-wide-significant hits (*P* <5 × 10^−8^) are indicated in red. Additional file 4: Figure S4. Annotation of rs9485370 and rs60705924 by their effect on regulatory motifs according to the HaploREG database. A = rs9485370; B = rs60705924. Additional file 5: Table S1. Associations between genome-wide-significant SNPs and breast cancer risk in the Breast Cancer Association Consortium (BCAC). Additional file 6: Table S2. Replication analysis of SNPs identified by MODE, per mammographic density phenotype.

## Abbreviations

AREG: amphiregulin
BCAC: Breast Cancer Association Consortium
BMI: body mass index
CCDC170: coiled-coil domain containing 170
ENCODE: Encyclopedia of DNA Elements
ER: estrogen receptor
ESR1: estrogen receptor 1
GWAS: genome-wide association study
HMEC: human mammary epithelial cells
HWE: Hardy–Weinberg equilibrium
iCOGS: Ilumina iSelect genotyping array of the Collaborative Oncological Gene-environment Study
IGF1: insulin-like growth factor 1
IL-1: interleukin-1
IMPUTEv2: IMPUTE version 2
KARMA: KARolinska MAmmography project for risk prediction of breast cancer
LD: linkage disequilibrium
LIBRO-1: Linné-bröst 1
LSP1: lymphocyte- specific protein 1
MAF: minor allele frequency
MAPK8/JNK: mitogen-activated protein kinase 8/c-Jun N-terminal kinase
MD: mammographic density
MKL1: MKL/myocardin-like protein 1
MLO: mediolateral oblique
MODE: the Marker of Density consortium
NFkB: nuclear factor kappa B
OR: odds ratio
PRDM6: PR domain containing 6
QC: quality control
QQ: quantile-quantile
SASBAC: the Singapore and Sweden Breast Cancer study
SGSM3: small G protein signaling modulator 3
SNP: single nucleotide polymorphism
TAB2: TGF-beta-activated kinase 1/MAP3K7-binding protein 2
TMEM184B: transmembrane protein 184b
TNNT3: troponin T type 3
ZNF365: zinc finger protein 365
