# Supplementary material for: Identification of two novel mammographic density loci at 6Q25.1
Source: Breast Cancer Res. 2015 Jun 3;17(1):75. doi: 10.1186/s13058-015-0591-2 (PMC4501298; doi:10.1186/s13058-015-0591-2)
Supplement: Additional file 1: Figure S1. — Distributions of mammographic density phenotypes, stratified by study. Distributions of mammographic measures before (A) and after transformation (B). Percent density in %; absolute dense tissue in cm3 (KARMA) and cm2 (SASBAC and LIBRO-1); absolute nondense tissue in cm3 (KARMA) and cm2 (SASBAC and LIBRO-1). Volumetric mammographic measures were log-transformed (KARMA) and area-based mammographic measures were square-root-transformed (SASBAC and LIBRO-1) prior to analyses. [file 13058_2015_591_MOESM1_ESM.docx]

**Figure S1.**

**A**

**Figure S1.** *continued*

**B**
