# Supplementary figures and images for: Identification of two novel mammographic density loci at 6Q25.1
Source: Breast Cancer Res. 2015 Jun 3;17(1):75. doi: 10.1186/s13058-015-0591-2 (PMC4501298; doi:10.1186/s13058-015-0591-2)

**Figure S2.**

**A B**

**
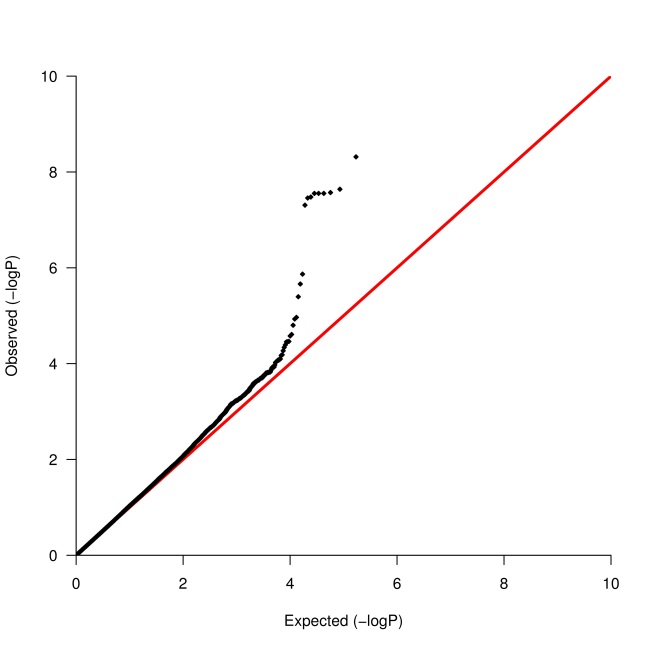

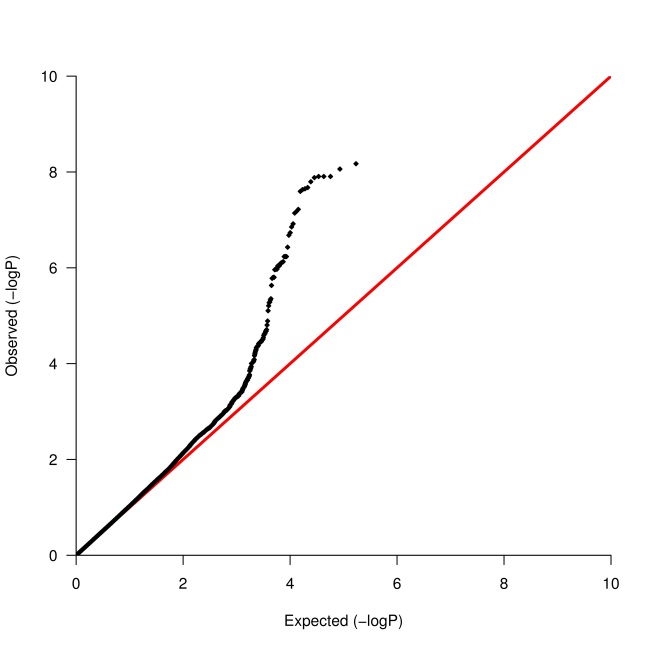
**

**C**

**
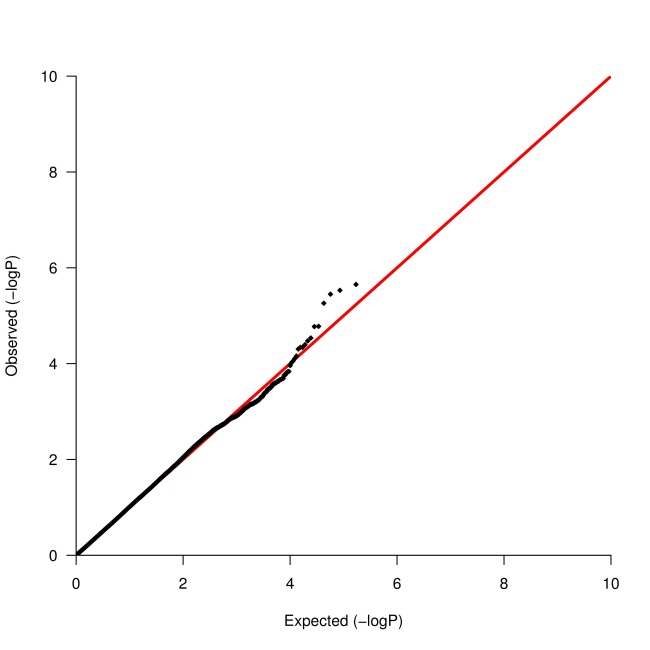
**

Supplement: Additional file 2: Figure S2. — Quantile-quantile (QQ) plots, per mammographic density phenotype. A = percent density; B = absolute dense tissue; C = absolute nondense tissue. The observed P values based the on meta-analysis of KARMA, SASBAC and LIBRO-1 are plotted against the expected distribution of P values under the null distribution. [file 13058_2015_591_MOESM2_ESM.docx]

**Figure S3.**

**A**

**
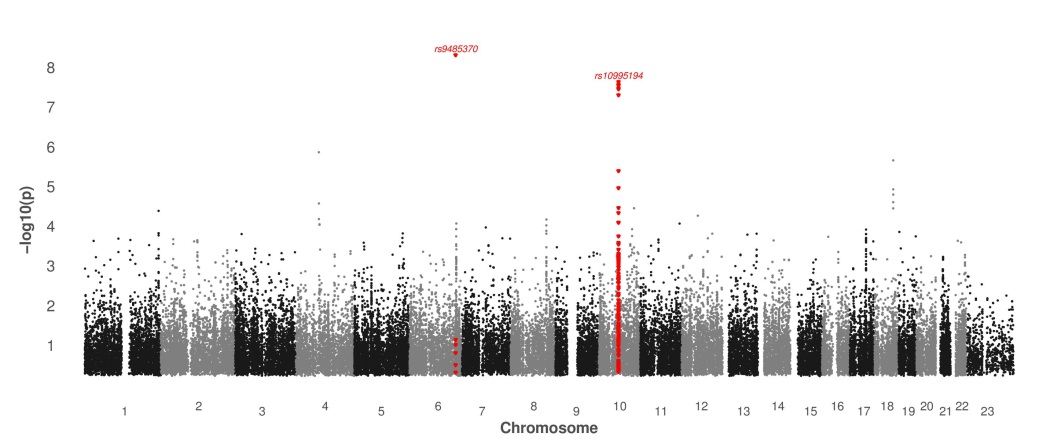
**

**Figure S3.** *continued*

**B**

**
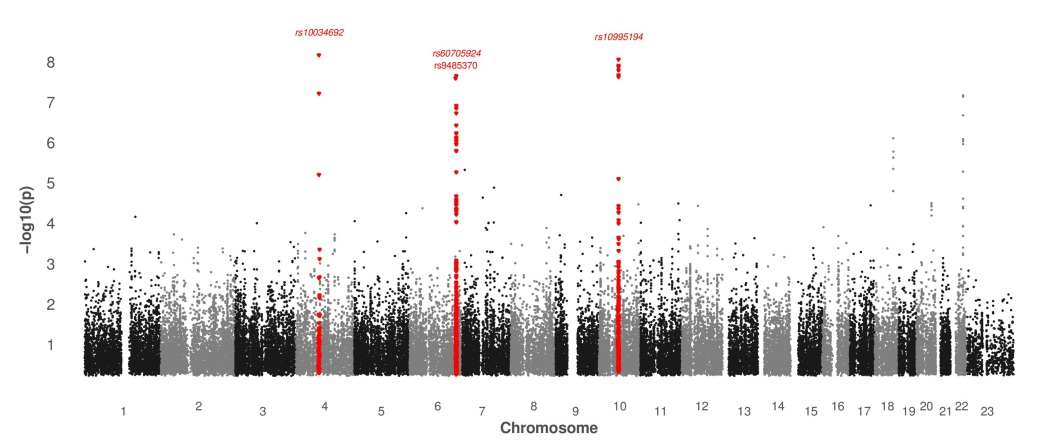
**

**Figure S3.** *continued*

**C**

**
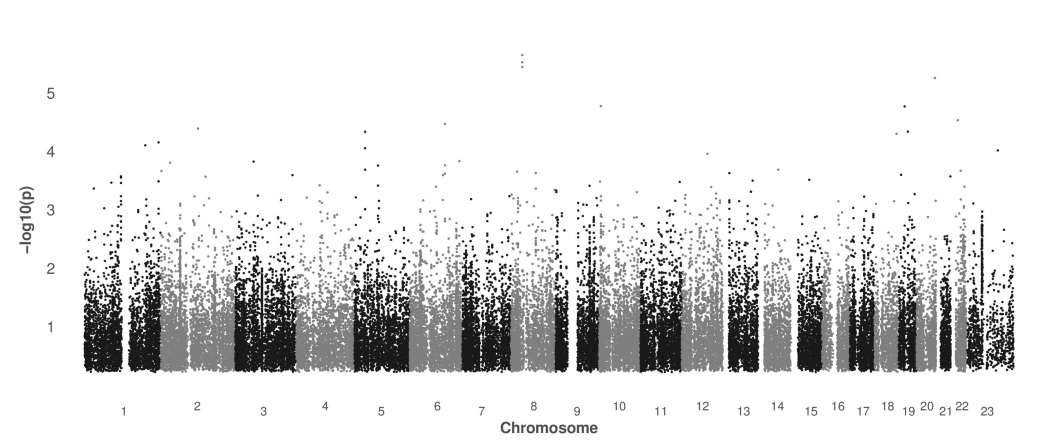
**

Supplement: Additional file 3: Figure S3. — Manhattan plots of the combined association results, per mammographic density phenotype. A = percent density; B = absolute dense tissue; C = absolute nondense tissue. The –log10 (P) values are plotted against chromosomal base-pair position. Genome-wide-significant hits (P <5 × 10^−8^) are indicated in red. [file 13058_2015_591_MOESM3_ESM.docx]

**Figure S4.**

**A**


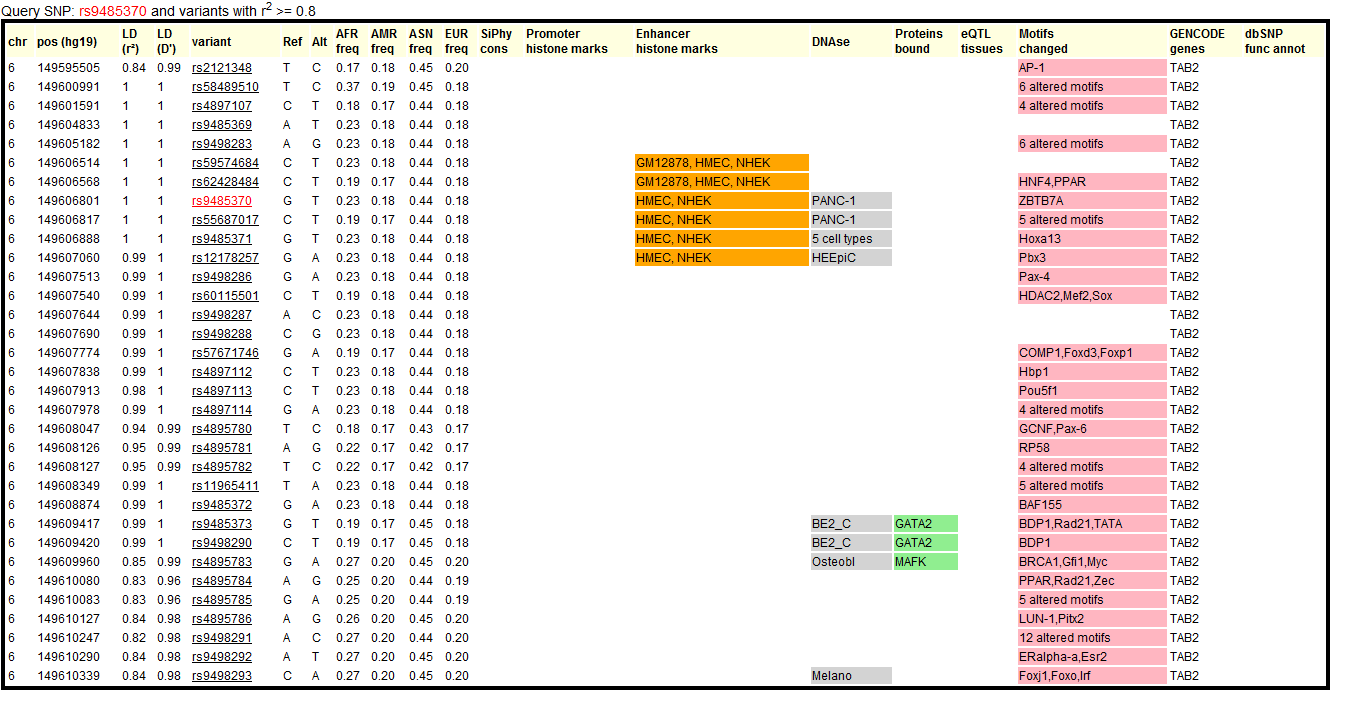


**Figure S4.** *continued*

**B**


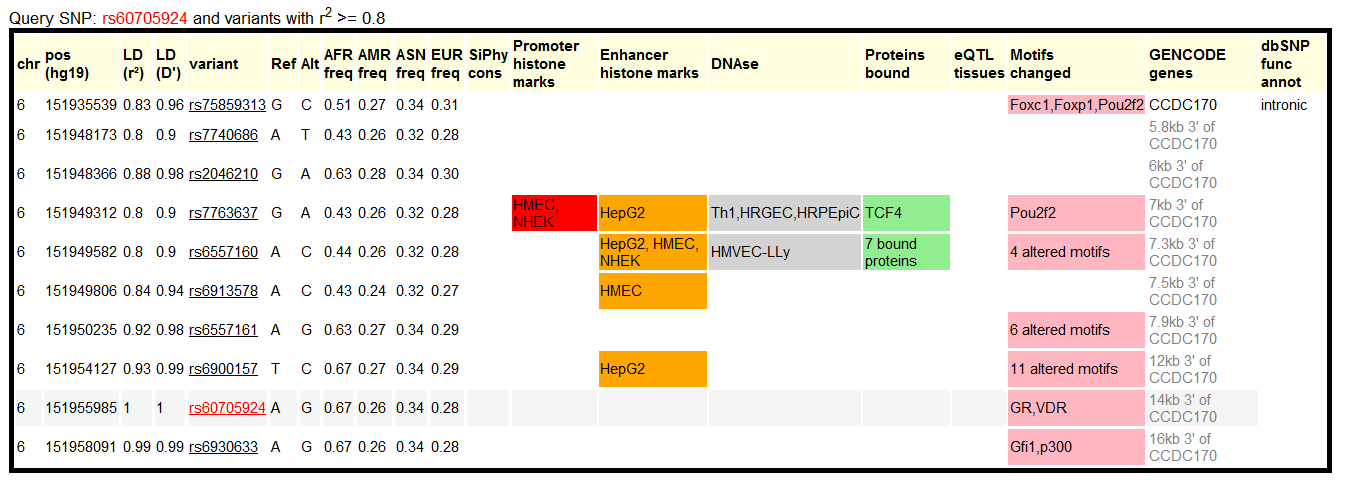

Supplement: Additional file 4: Figure S4. — Annotation of rs9485370 and rs60705924 by their effect on regulatory motifs according to the HaploREG database. A = rs9485370; B = rs60705924. [file 13058_2015_591_MOESM4_ESM.docx]
