# Supplementary material for: Identification of two novel mammographic density loci at 6Q25.1
Source: Breast Cancer Res. 2015 Jun 3;17(1):75. doi: 10.1186/s13058-015-0591-2 (PMC4501298; doi:10.1186/s13058-015-0591-2)
Supplement: Additional file 5: Table S1. — Associations between genome-wide-significant SNPs and breast cancer risk in the Breast Cancer Association Consortium (BCAC). [file 13058_2015_591_MOESM5_ESM.docx]

**Table S1.** Associations between genome-wide significant SNPs and breast cancer risk in the breast cancer association consortium (BCAC).

| **CHR** | **SNP** | **Genes** | **Alleles** | **Ethnicity** | **N** | **MAF ^1^** | **Overall** | | **ER +** | | **ER-** | |
| --- | --- | --- | --- | --- | --- | --- | --- | --- | --- | --- | --- | --- |
|  |  |  |  |  |  |  | **OR** | ***P*** | **OR** | ***P*** | **OR** | ***P*** |
| 6 | rs9485370 | *TAB2* | G/T | European | 91,767 | 0.18 | 0.96 | 1.4x10^-3^ | 0.95 | 5.7x10^-4^ | 0.95 | 0.05 |
|  |  |  |  | Asian | 12,893 | 0.42 | 0.89 | 7.4x10^-6^ | 0.91 | 3.4x10^-3^ | 0.89 | 4.7x10^-3^ |
|  |  |  |  | African-American | 2,048 | 0.22 | 0.96 | 0.63 | 0.88 | 0.41 | 0.97 | 0.87 |
| 6 | rs60705924 | *CCDC170/ESR1* | A/G | European | 91,767 | 0.32 | 1.08 | 1.9x10^-13^ | 1.05 | 2.1x10^-5^ | 1.15 | 4.3x10^-12^ |
|  |  |  |  | Asian | 12,893 | 0.37 | 1.28 | 4.3x10^-20^ | 1.23 | 1.2x10^-10^ | 1.43 | 2.0x10^-17^ |
|  |  |  |  | African-American | 2,048 | 0.66 | 1.16 | 0.03 | 1.01 | 0.95 | 1.10 | 0.59 |

Abbreviations: CHR = chromosome; SNP = single nucleotide polymorphism; Alleles: Major allele (reference allele)/Minor allele (effect allele); MAF = minor allele frequencies as observed in BCAC populations; Overall = all breast cancers regardless of estrogen receptor status; ER+ = estrogen receptor positive cancers; ER- = estrogen receptor negative cancers; OR = odds ratio per minor allele increase. Genes refer to genes and nearby genes. ^1^ Number of breast cancer cases and controls per study sample: Europeans (N cases = 48,155; N controls = 43,612), Asians (N cases = 6,269 and N controls = 6,624); African-American (N cases = 1,116; N controls = 932).
