# Supplementary material for: Identification of two novel mammographic density loci at 6Q25.1
Source: Breast Cancer Res. 2015 Jun 3;17(1):75. doi: 10.1186/s13058-015-0591-2 (PMC4501298; doi:10.1186/s13058-015-0591-2)
Supplement: Additional file 6: Table S2. — Replication analysis of SNPs identified by MODE, per mammographic density phenotype. [file 13058_2015_591_MOESM6_ESM.docx]

**Table S2.** Replication analysis of SNPs identified by MODE, per mammographic density phenotype.

| **CHR** | **SNP** | **BP** | **Gene** | **Alleles ^1^** | **MAF** | **MODE** |  | **KARMA** |  | **LIBRO-1** |  | ***P* overall** | ***P* het** |
| --- | --- | --- | --- | --- | --- | --- | --- | --- | --- | --- | --- | --- | --- |
|  |  |  |  |  |  | **beta (se)** | ***P*** | **beta (se)** | ***P*** | **beta (se)** | ***P*** |  |  |
| Percent density | | | | | | | | | | | | | |
| 5 | rs186749 | 122482204 | *PRDM6* | G/A | 0.34 | 0.10 (0.02) | 2.5x10^-9^ | 0.03 (0.02) | 0.15 | 0.04 (0.03) | 0.13 | 0.04 | 0.81 |
| 8 | rs7816345 | 36965267 | *N/A* | C/T | 0.16 | 0.08 (0.02) | 4.7x10^-8^ | 0.01 (0.01) | 0.30 | 0.02 (0.05) | 0.71 | 0.31 | 0.71 |
| 22 | rs7289126 | 36958252 | *TMEM184B* | C/A | 0.45 | -0.10 (0.02) | 4.7x10^-9^ | -0.02 (0.01) | 0.03 | 0.01 (0.03) | 0.82 | 0.14 | 0.13 |
| Absolute dense | | | | | | | | | | | | | |
| 4 | rs10034692 | 75638651 | *AREG* | A/G | 0.29 | -0.16 (0.03) | 2.1x10^-10^ | -0.04 (0.01) | 9.6x10^-5^ | -0.14 (0.04) | 5.5x10^-4^ | 3.0x10^-7^ | 0.87 |
| 6 | rs12665607 | 151988322 | *ESR1* | T/A | 0.08 | 0.17 (0.04) | 1.7x10^-8^ | 0.08 (0.02) | 7.2x10^-5^ | 0.08 (0.08) | 0.28 | 2.4x10^-4^ | 0.09 |
| 10 | rs10995190 | 63948688 | *ZNF365* | G/A | 0.14 | -0.24 (0.03) | 1.5x10^-16^ | -0.06 (0.01) | 1.8x10^-6^ | -0.16 (0.05) | 4.7x10^-3^ | 7.3x10^-8^ | 0.39 |
| 11 | rs3817198 | 1865582 | *LSP1* | T/C | 0.32 | 0.14 (0.03) | 9.7x10^-11^ | 0.004 (0.01) | 0.71 | 0.06 (0.04) | 0.16 | 0.25 | 0.41 |
| 12 | rs703556 | 101536024 | *IGF1* | A/G | 0.04 | -0.41 (0.08) | 3.7x10^-10^ | -0.19 (0.06) | 9.1x10^-4^ | -0.13 (0.07) | 0.06 | 1.6x10^-4^ | 0.51 |
| 22 | rs7289126 | 36958252 | *TMEM184B* | C/A | 0.45 | -0.11 (0.02) | 2.8x10^-8^ | -0.02 (0.01) | 0.08 | 0.02 (0.04) | 0.69 | 0.30 | 0.16 |
| 22 | rs17001868 | 39108177 | *SGSM3, MKL1* | A/C | 0.13 | -0.18 (0.03) | 2.3x10^-13^ | -0.03 (0.01) | 0.05 | -0.12 (0.05) | 0.02 | 2.8x10^-3^ | 0.53 |
| Absolute nondense | | | | | | | | | | | | | |
| 8 | rs7816345 | 36965267 | *N/A* | C/T | 0.16 | -0.24 (0.03) | 2.4x10^-23^ | -0.04 (0.01) | 1.2x10^-3^ | -0.09 (0.05) | 0.06 | 2.4x10^-4^ | 0.57 |

^1^ Major allele (reference allele)/Minor allele (effect allele). Abbreviations: CHR = chromosome; SNP = single nucleotide polymorphism; BP = base pair position (Build 37); MAF = minor allele frequencies as in the 1000 Genomes project (EUR sample); beta = beta coefficients per minor allele increase; se = standard error; P overall = p values combined meta-analysis KARMA and LIBRO-1; P het = p values for heterogeneity between KARMA and LIBRO-1 study. Genes refer to genes and nearby genes.
